# Associations of Pregnancy Outcomes and PM_2.5_ in a National Canadian Study

**DOI:** 10.1289/ehp.1408995

**Published:** 2015-06-19

**Authors:** David M. Stieb, Li Chen, Bernardo S. Beckerman, Michael Jerrett, Daniel L. Crouse, D. Walter Rasugu Omariba, Paul A. Peters, Aaron van Donkelaar, Randall V. Martin, Richard T. Burnett, Nicolas L. Gilbert, Michael Tjepkema, Shiliang Liu, Rose M. Dugandzic

**Affiliations:** 1 Population Studies Division, Health Canada, Vancouver, British Columbia, Canada; 2 Population Studies Division, Health Canada, Ottawa, Ontario, Canada; 3 Geographic Information Health and Exposure Science Laboratory (GIS HEAL), School of Public Health, University of California, Berkeley, Berkeley, California, USA; 4 Department of Environmental Health Sciences, Fielding School of Public Health, University of California, Los Angeles, Los Angeles, California, USA; 5 Department of Sociology, University of New Brunswick, Fredericton, New Brunswick, Canada; 6 Special Surveys Division, Statistics Canada, Ottawa, Ontario, Canada; 7 Department of Physics and Atmospheric Science, Dalhousie University, Halifax, Nova Scotia, Canada; 8 Harvard-Smithsonian Center for Astrophysics, Cambridge, Massachusetts, USA; 9 Vaccine and Immunization Program Surveillance Division, Public Health Agency of Canada, Ottawa, Canada; 10 Health Analysis Division, Statistics Canada, Ottawa, Ontario, Canada; 11 Maternal, Child and Youth Health, Surveillance and Epidemiology Division, Public Health Agency of Canada, Ottawa, Ontario, Canada; 12 Air Health Science Division, Health Canada, Ottawa, Ontario, Canada

## Abstract

**Background:**

Numerous studies have examined associations between air pollution and pregnancy outcomes, but most have been restricted to urban populations living near monitors.

**Objectives:**

We examined the association between pregnancy outcomes and fine particulate matter in a large national study including urban and rural areas.

**Methods:**

Analyses were based on approximately 3 million singleton live births in Canada between 1999 and 2008. Exposures to PM_2.5_ (particles of median aerodynamic diameter ≤ 2.5 μm) were assigned by mapping the mother’s postal code to a monthly surface based on a national land use regression model that incorporated observations from fixed-site monitoring stations and satellite-derived estimates of PM_2.5_. Generalized estimating equations were used to examine the association between PM_2.5_ and preterm birth (gestational age < 37 weeks), term low birth weight (< 2,500 g), small for gestational age (SGA; < 10th percentile of birth weight for gestational age), and term birth weight, adjusting for individual covariates and neighborhood socioeconomic status (SES).

**Results:**

In fully adjusted models, a 10-μg/m^3^ increase in PM_2.5_ over the entire pregnancy was associated with SGA (odds ratio = 1.04; 95% CI 1.01, 1.07) and reduced term birth weight (–20.5 g; 95% CI –24.7, –16.4). Associations varied across subgroups based on maternal place of birth and period (1999–2003 vs. 2004–2008).

**Conclusions:**

This study, based on approximately 3 million births across Canada and employing PM_2.5_ estimates from a national spatiotemporal model, provides further evidence linking PM_2.5_ and pregnancy outcomes.

**Citation:**

Stieb DM, Chen L, Beckerman BS, Jerrett M, Crouse DL, Omariba DW, Peters PA, van Donkelaar A, Martin RV, Burnett RT, Gilbert NL, Tjepkema M, Liu S, Dugandzic RM. 2016. Associations of pregnancy outcomes and PM_2.5_ in a National Canadian Study. Environ Health Perspect 124:243–249; http://dx.doi.org/10.1289/ehp.1408995

## Introduction

Numerous studies have examined the association between air pollution and pregnancy outcomes. Meta-analyses and pooled multi-center analyses suggest that particulate matter is associated with low birth weight and preterm birth, although there is heterogeneity among centers (Dadvand et al. 2013; Stieb et al. 2012). Most studies have been based on exposure estimates from fixed-site monitoring data, and therefore have been restricted to urban populations living in the vicinity of monitoring stations (Stieb et al. 2012). However, population coverage of ground-based monitoring is low; Guay et al. (2011) found that the proportion of National Population Health Survey participants in Canada living in a census subdivision containing an air pollution monitor was at best 41% among the various pollutants considered. Estimates of particulate matter concentrations from models and/or satellite observations have made it possible to extend analyses of effects to large national studies comprising both urban and rural areas (Crouse et al. 2012; Lim et al. 2012). To this point, however, few analyses have been reported of model/satellite-based exposure estimates and pregnancy outcomes (Fleischer et al. 2014; Hyder et al. 2014; Kloog et al. 2012).

In this study we employed estimates of fine particulate matter (median aerodynamic diameter ≤ 2.5 μm; PM_2.5_) from a spatiotemporal model using ground measurements and satellite-based estimates and applied these to approximately 3 million births across Canada between 1999 and 2008. We examined preterm birth, term low birth weight (LBW), and small for gestational age (SGA) as binary variables, and term birth weight as a continuous variable.

## Methods

*Pregnancy outcome data.* Data on all singleton live births between 1999 and 2008 were accessed through Statistics Canada (2015c) after obtaining approval from Health Canada’s Research Ethics Board. Birth records included data on infant sex, date of birth, gestational age, birth weight, birth order, number of stillborn (if multiple birth), postal code of maternal place of residence at child’s birth, maternal age at child’s birth, marital status, total number of liveborn and stillborn (ever), province/country of mother and father’s birth, and mother’s education level in years (Quebec only). Data on maternal behaviors including smoking and alcohol consumption were not available. Pregnancy outcomes under study were preterm birth (gestational age < 37 weeks), term LBW (< 2,500 g), SGA (< 10 percentile of birth weight for gestational age) (Kramer et al. 2001), and term birth weight as a continuous variable.

*Geocoding and socioeconomic status.* The Postal Code Conversion File Plus (PCCF+) (82F0086X; http://www5.statcan.gc.ca/olc-cel/olc.action?objId=82F0086X&objType=2&lang=en&limit=0) was used to geocode birth records using the maternal postal code in order to obtain Statistics Canada standard geographic identifiers. In urban Canada (75–80% of the population), postal codes generally refer to a small geographic area containing on average 30 people. Each postal code is represented spatially by a representative point or points. In urban areas, it is most often located at the mid-point along a block-face portion that generally corresponds to one side of a road. For apartment buildings it is often the location of the building. For rural Canada, postal codes can cover a large geographic area with as many as 1,100 people, encompassing more than one census dissemination area. For these cases, postal code representative points are randomly allocated using a population-weighted file from Statistics Canada [Postal CodeOM Conversion File Plus (PCCF+), June 2013; http://www5.statcan.gc.ca/olc-cel/olc.action?ObjId=82F0086X2014001&ObjType=46&lang=en], such that the probability of a given dissemination area (DA) centroid being used reflects the spatial distribution of the underlying population. Postal codes were considered rural if the second character was zero. Using geocoded birth records, neighborhood-level socioeconomic status (SES) variables were calculated at the DA level using census data, including proportion of individuals ≥ 15 years of age who were unemployed, proportion of individuals ≥ 15 years of age in the lowest income quintile, and proportion of females ≥ 25 years of age with postsecondary education (Crouse et al. 2012; Dadvand et al. 2013). Variables were calculated based on the census closest to the date of birth (2001 or 2006) (Statistics Canada 2015a). There were 52,993 and 54,626 dissemination areas in the 2001 and 2006 censuses respectively, each with a population of approximately 400–700 people.

*PM_2.5_ data.* PM_2.5_ exposures were assigned by mapping the mother’s six-character postal code to a monthly surface based on a North American land use regression (LUR) model that incorporated observations from fixed-site monitoring stations and satellite-derived estimates of PM_2.5_. Exposures were estimated for the entire duration of pregnancy, by trimester, and by gestational month.

Using the same two-stage methods as those described by Beckerman et al. (2013), we created a predictive spatiotemporal exposure model for ambient PM_2.5_ for Canada by combining observed PM_2.5_ levels with observations from the contiguous United States. These observations were combined to help stabilize the variability likely to be predicted by the small Canadian data set. In Canada, there were 241 sites over a very large landmass, whereas the U.S. data set had a total of 1,464 sites. There was a concern that the limited density of observations in Canada would reduce our ability to generate defensible predictive estimates. Given the adjacency of the observations in the United Staters, we determined that they could bolster the predictive capacity of a Canadian model.

During the first stage of modeling, a machine learning method, known as the deletion/substitution/addition (DSA) algorithm, was implemented to create a LUR model, as described by Beckerman et al. (2013). Variables describing square of open (undeveloped) space within 200 m of a location and PM_2.5_ concentration estimated from remote sensing (squared and cubed) were chosen by the DSA algorithm, for the LUR model as the most predictive variables, using cross-validation selection techniques. Additionally, an indicator for the Canadian data set was interacted with the remote sensing variable to provide a small marginal adjustment to the remote sensing contribution to the prediction. The polynomial terms served to reduce bias in the remote sensing estimates because they did not use any ground data to calibrate the predicted levels. The polynomial term on open space was selected by DSA because it predicted best and described the functional form of the relationship with observed PM_2.5_ levels. The prediction results were very similar to those reported by Beckerman et al. (2013) (see Supplemental Material, Table S1). For Canada, the LUR model described 59% of the observed variability in the mean as measured by the CV normalized pseudo-*R*^2^ based on v-fold CV prediction error. However, there was significant residual variability as the non-normalized CV pseudo-*R*^2^ was 26%. In the second stage, the Bayesian maximum entropy interpolation method (Christakos 1990) was used to create a spatiotemporal prediction model of the space–time residuals from the LUR model that were added to the LUR prediction estimates. This method (described in more detail by Beckerman et al. 2013), produced a final model with a CV *R*^2^ of 0.36. CV estimates were based on 1,436 (10%) randomly selected leave-out observations from 22 monitoring sites. This model appeared less predictive than the U.S. model (CV *R*^2^ = 0.79); however, the poor fit was partly driven by a small number of outlying observations (see Supplemental Material, Figure S1) and removing them improved the model prediction (CV *R*^2^ = 0.53).

As a sensitivity analysis, we also employed monthly average concentrations from ground-based monitors for 24 cities with at least 85% complete monthly data. The total population of these cities based on the 2006 census was approximately 11,500,000, or about one-third of the Canadian population.

*Statistical analysis.* We used a similar approach to that employed in the recent International Collaboration on Air Pollution and Pregnancy Outcomes (ICAPPO) multi-center analysis (Dadvand et al. 2013), reporting unadjusted results, and incrementally adding adjustments for socioeconomic status, and maternal and infant characteristics. Generalized estimating equations (GEE) were used to examine the association between air pollution and preterm birth, term LBW, SGA, and term birth weight, adjusting for covariates including infant sex, gestational age, parental age and marital status, parity, urban/rural place of residence, place of birth of parents (within/outside Canada), season [winter (January–March), spring (April–June), summer (July–September) and fall (October–December)], year of birth, and DA proportions of individuals ≥ 15 years of age who were unemployed, of individuals ≥ 15 years of age in the lowest income quintile, of females ≥ 25 years of age with postsecondary education and of individuals who were visible minority. Visible minority groups are those defined by the Canadian Employment Equity Act, and classification of individuals is based on response to census questions pertaining to self-identified population and aboriginal group (Statistics Canada 2015b). Because some provinces and territories had few births, we adjusted for location of mother’s place of residence based on six regional airsheds (see Supplemental Material, Figure S2) (J. Brook, Environment Canada, personal communication). We accounted for clustering of observations by DA by treating births from the same DA as repeated subjects in the GEE analysis. Subgroup analyses were conducted based on maternal place of birth (within vs. outside Canada), urban versus rural place of residence, neighborhood SES, and study period (1999–2003, 2004–2008). Results were considered statistically significant if *p*-value < 0.05. All analyses were conducted using SAS version 9.3 (SAS Institute Inc.).

## Results

During the study period there were 3,104,090 live births. Of these, 3,061,155 (98.6%) could be mapped to PM_2.5_ exposures, of which 2,969,380 were singletons (in accordance with Statistics Canada disclosure rules, all frequencies were randomly rounded to base five). After further excluding births with missing covariate data, analyses of preterm birth, term LBW and birth weight, and SGA were based on up to 2,966,705, 2,781,940, and 2,965,440 births, respectively. The overall prevalence of preterm birth was 6.23%, of term LBW 1.57%, and SGA 8.31%. Mean assigned PM_2.5_ exposure over the entire pregnancy was 8.4 μg/m^3^ and interquartile range (IQR) was 3.6 μg/m^3^ (Table 1). Mean exposures by trimester and month were similar, but there was somewhat greater variability compared with exposure over the entire pregnancy, reflected by greater standard deviations and IQRs. Entire pregnancy exposures were highly correlated with trimester and monthly exposure periods (Spearman’s *r* = 0.85–0.91 and 0.74–0.83, respectively) (see Supplemental Material, Table S2). Prevalence of each outcome and mean PM_2.5_ exposure by infant, maternal, and neighborhood characteristics are shown in Table 2. Appreciable differences in both outcome prevalence and exposure were noted by infant, maternal, and neighborhood characteristics. In particular, there was a time trend of reduced PM_2.5_, but not of pregnancy outcome, between 1999 and 2008. There was also a consistent trend of increased prevalence of adverse pregnancy outcome in neighborhoods in the lowest tertile of SES indicators, but there was no consistent trend in PM_2.5_ exposure in relation to SES: There was no trend in PM_2.5_ exposure by tertile of percent unemployed; PM_2.5_ exposure was higher in the highest tertile of percent low income; and there was a trend of higher PM_2.5_ exposure with increasing percent of females who completed postsecondary education. Gradients in neighborhood SES variables were consistent with those in individual-level maternal education in Quebec, where individual level data on maternal education were available: Compared with mothers who had lower educational attainment, a larger percentage of mothers with higher educational attainment lived in DAs containing the highest percentage of females who had completed postsecondary education, the lowest percentage of individuals in the lowest income quintile, and the lowest percentage of unemployed individuals. Conversely, compared with mothers with higher educational attainment, a larger percentage of mothers with lower educational attainment lived in DAs containing the lowest percentage of females who had completed postsecondary education, the highest percentage of individuals in the lowest income quintile, and the highest percentage of unemployed individuals (see Supplemental Material, Table S3).

**Table 1:** Summary of estimated PM_2.5_ exposures by exposure period (μg/m^3^).

Period	*n*	Mean	Standard deviation	5th Percentile	Median	95th Percentile	Interquartile range
Entire pregnancy	2,966,705	8.43	2.40	4.61	8.44	12.42	3.58
Trimester 1	2,966,700	8.50	2.78	4.29	8.36	13.20	4.01
Trimester 2	2,966,705	8.44	2.77	4.26	8.30	13.14	3.99
Trimester 3	2,954,665	8.36	2.75	4.22	8.21	13.00	3.95
Month 1	2,966,695	8.51	3.11	4.08	8.20	13.89	4.35
Month 2	2,966,695	8.50	3.11	4.08	8.18	13.88	4.33
Month 3	2,966,700	8.48	3.11	4.06	8.17	13.87	4.34
Month 4	2,966,700	8.47	3.11	4.05	8.15	13.84	4.32
Month 5	2,966,700	8.45	3.10	4.04	8.13	13.81	4.31
Month 6	2,960,860	8.42	3.09	4.02	8.10	13.75	4.30
Month 7	2,951,295	8.39	3.09	4.01	8.06	13.71	4.28
Month 8	2,900,745	8.37	3.08	4.00	8.03	13.70	4.26
Month 9	2,086,560	8.33	3.08	3.98	7.99	13.66	4.27

**Table 2:** Prevalence of pregnancy outcomes and mean PM_2.5_ exposures over the entire pregnancy by infant, maternal, and neighborhood characteristics.

Variable	Preterm birth [*n* (%)]	Term LBW [*n* (%)]	SGA [*n* (%)]	Mean PM_2.5_ (μg/m^3^)
Sex
Male	101,495 (6.67)	17,925 (1.26)	129,525 (8.51)	8.4
Female	83,270 (5.77)	25,640 (1.88)	117,030 (8.11)	8.4
Unknown	NR^*a*^	0 (0.0)	0 (0.0)	8.4
Maternal age (years)
< 18	3,190 (8.08)	700 (1.93)	3,850 (9.76)	7.6
18–29	105,190 (6.13)	25,810 (1.60)	149,705 (8.73)	8.3
30–39	72,125 (6.20)	16,015 (1.47)	88,390 (7.60)	8.6
≥ 40	4,240 (8.72)	1,035 (2.33)	4,595 (9.46)	8.7
Unknown	20 (11.76)	NR	15 (8.82)	8.8
Marital status
Single	53,960 (6.98)	13,150 (1.83)	71,900 (9.30)	8.2
Married	105,620 (5.70)	24,685 (1.41)	143,540 (7.74)	8.5
Widowed	190 (8.09)	40 (1.85)	220 (9.38)	8.8
Divorced	2,505 (7.54)	575 (1.87)	3,000 (9.03)	8.2
Separated	970 (8.51)	215 (2.06)	1,050 (9.21)	7.0
Unknown	21,520 (7.37)	4,900 (1.81)	26,845 (9.20)	8.4
Maternal place of birth
Canadian born	136,690 (6.26)	28,420 (1.39)	161,465 (7.39)	8.1
Not Canadian born	44,920 (6.08)	14,370 (2.07)	80,835 (10.95)	9.4
Unknown	3,155 (7.26)	775 (1.92)	4,255 (9.80)	10.3
Maternal place of residence
Urban	152,035 (6.24)	36,930 (1.62)	208,610 (8.57)	8.8
Rural	32,730 (6.15)	6,635 (1.33)	37,945 (7.14)	6.6
Parity
1	91,230 (6.91)	23,765 (1.93)	139,055 (10.54)	8.5
2	54,345 (5.26)	11,870 (1.21)	66,940 (6.48)	8.5
≥ 3	38,085 (6.39)	7,680 (1.38)	39,195 (6.58)	8.2
Unknown	1,105 (6.30)	250 (1.52)	1,365 (7.79)	6.3
Maternal province of residence
Newfoundland and Labrador	2,760 (6.77)	490 (1.29)	2,775 (6.81)	5.0
Prince Edward Island	640 (5.18)	140 (1.20)	840 (6.80)	5.3
Nova Scotia	4,705 (6.19)	1,135 (1.59)	6,165 (8.12)	6.1
New Brunswick	3,850 (6.10)	795 (1.34)	4,765 (7.56)	5.4
Quebec	42,715 (6.26)	9,815 (1.54)	55,825 (8.19)	9.5
Ontario	67,715 (6.01)	18,100 (1.71)	98,670 (8.76)	9.6
Manitoba	8,465 (6.66)	1,610 (1.36)	9,740 (7.67)	6.2
Saskatchewan	6,320 (6.05)	1,405 (1.43)	7,795 (7.46)	6.2
Alberta	26,070 (7.05)	5,505 (1.60)	32,000 (8.66)	7.9
British Columbia	21,465 (5.90)	4,560 (1.33)	27,930 (7.68)	6.5
Yukon	15 (11.11)	NR	5 (3.85)	5.2
NWT	20 (6.45)	NR	20 (6.45)	3.9
Nunavut	20 (11.11)	10 (6.25)	25 (14.29)	5.9
Unknown	5 (33.33)	0 (0.00)	NR	7.9
Birth year
1999	3,125 (6.45)	710 (1.57)	4,245 (8.77)	8.8
2000	19,340 (6.20)	4,410 (1.51)	25,375 (8.14)	9.0
2001	18,995 (5.97)	4,610 (1.54)	26,465 (8.33)	8.9
2002	18,745 (6.00)	4,580 (1.56)	26,000 (8.33)	8.8
2003	17,185 (6.2)	4,080 (1.57)	23,135 (8.35)	9.1
2004	20,305 (6.39)	4,615 (1.55)	25,770 (8.12)	8.5
2005	20,695 (6.30)	4,820 (1.57)	27,525 (8.39)	8.6
2006	21,620 (6.36)	5,245 (1.65)	28,905 (8.51)	8.2
2007	21,740 (6.20)	5,175 (1.57)	29,615 (8.46)	7.5
2008	23,015 (6.35)	5,320 (1.57)	29,520 (8.14)	7.6
1999–2003	77,390 (6.10)	18,390 (1.54)	103,220 (8.3)	8.95
2004–2008	107,375 (6.32)	25,175 (1.58)	141,335 (8.32)	8.05
Season
Spring	46,690 (6.18)	10,550 (1.49)	60,355 (8.00)	8.1
Summer	45,980 (6.02)	11,220 (1.56)	64,170 (8.40)	8.6
Fall	47,010 (6.35)	11,195 (1.61)	63,140 (8.53)	8.4
Winter	45,085 (6.38)	10,600 (1.60)	58,895 (8.34)	8.7
Percent unemployed (age ≥ 15 years)
1st tertile (≤ 4.6%)	58,785 (5.98)	13,020 (1.41)	75,945 (7.72)	8.4
2nd tertile (4.61–8.22%)	60,380 (6.14)	14,255 (1.54)	81,180 (8.26)	8.5
3rd tertile (> 8.22%)	64,810 (6.56)	16,120 (1.75)	88,535 (8.97)	8.4
Unknown	790 (6.55)	170 (1.51)	900 (7.47)	7.9
Percent in lowest income quintile (age ≥ 15 years)
1st tertile (≤ 9.25%)	57,235 (5.82)	11,890 (1.28)	70,080 (7.12)	8.2
2nd tertile (9.26–20.18%)	59,970 (6.10)	13,970 (1.51)	80,320 (8.17)	8.3
3rd tertile (> 20.18%)	66,770 (6.76)	17,530 (1.90)	95,260 (9.65)	8.8
Unknown	790 (6.55)	175 (1.55)	895 (7.43)	7.9
Percent of females completed postsecondary education (age ≥ 25 years)
1st tertile (≤ 20.36%)	65,585 (6.66)	15,900 (1.73)	86,385 (8.78)	8.1
2nd tertile (20.37–28.47%)	61,085 (6.21)	14,610 (1.58)	82,850 (8.42)	8.5
3rd tertile (> 28.47%)	57,305 (5.81)	12,885 (1.39)	76,425 (7.75)	8.8
Unknown	790 (6.55)	170 (1.51)	895 (7.43)	7.9
Percent visible minority
1st tertile (≤ 2.04%)	60,480 (6.20)	12,575 (1.37)	71,415 (7.33)	7.3
2nd tertile (2.05–16.13%)	59,685 (6.08)	12,745 (1.38)	74,640 (7.60)	8.6
3rd tertile (> 16.13%)	62,640 (6.39)	17,825 (1.94)	98,260 (10.03)	9.4
Missing	1,960 (6.75)	415 (1.53)	2,240 (7.72)	7.3
Total	184,765 (6.23)	43,560 (1.57)	246,555 (8.31)	8.4
NWT, Northwest Territories. ^***a***^Not reported. In accordance with Statistics Canada disclosure rules, case counts of less than five were suppressed, and all frequencies were randomly rounded to base five. Statistical analyses employed unrounded data.				

**Figure 1:**
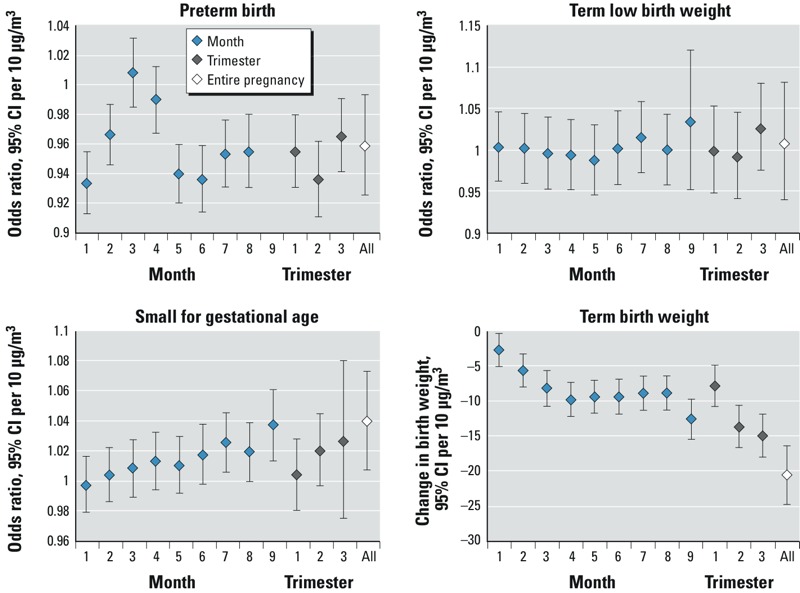
Associations of PM_2.5_ with pregnancy outcomes by exposure period. These were estimated using generalized estimating equations, accounting for clustering of observations by census dissemination area and adjusting for census dissemination area proportion of individuals ≥ 15 years of age who were unemployed (preterm birth model only), proportion of individuals ≥ 15 years of age in the lowest income quintile, and proportion of females ≥ 25 years of age with postsecondary education, maternal age and marital status, parity, urban/rural place of residence, airshed of maternal place of residence, place of birth of mother (within/outside Canada), year of birth, season of birth, and proportion of census dissemination area population who are visible minority; infant sex was also included in preterm birth, LBW, and birth weight models, and gestational age was also included in LBW and birth weight models. An estimate is not provided for preterm birth and exposure during the 9th month of gestation because only noncases would have been exposed during this time period.

Associations between PM_2.5_ and pregnancy outcomes based on exposures averaged over the entire pregnancy are shown in Table 3, by level of adjustment for covariates. PM_2.5_ exhibited a significant negative association with preterm birth in both unadjusted and fully adjusted models. SGA and term LBW exhibited significant positive associations, and term birth weight a significant negative association with PM_2.5_ in unadjusted models. Associations of PM_2.5_ with term LBW, SGA, and term birth weight were not sensitive to adjustment for neighborhood SES; however, they were all reduced substantially after adjustment for infant and maternal characteristics. Analysis by exposure period (Figure 1) revealed significant negative associations between PM_2.5_ and preterm birth for all exposure periods except months 2 and 3, neither of which were significant. Associations with term LBW were consistently null over all exposure periods. Late pregnancy exposures appeared to exhibit stronger associations with SGA and term birth weight. There was a very large but imprecise estimated reduction in birth weight [10-μg/m^3^ increase associated with reduction of –46.3 g; 95% confidence interval (CI): –74.6, –18.0] for a small number of births (22,805) with gestational age > 9 months. We did not include this in the figure because it obscured the results for all other exposure periods. The association with term birth weight based on exposure over the entire pregnancy was not sensitive to exclusion of these births (–21.4 g; 95% CI: –25.6, –17.1 vs. –20.5 g; 95% CI: –24.7, –16.4 including these births).

**Table 3:** Associations between PM_2.5_ over the entire pregnancy and pregnancy outcome, by level of adjustment (per 10 μg/m^3^).

Model	Preterm birth OR (95% CI)	Term LBW OR (95% CI)	SGA OR (95% CI)	Term birth weight β (95% CI)
Unadjusted	0.96 (0.93, 0.98)	1.51 (1.44, 1.57)	1.46 (1.43, 1.49)	–115.5 (–119.7, –111.3)
+ neighborhood SES^*a*^	0.97 (0.95, 1.00)	1.47 (1.41, 1.54)	1.41 (1.38, 1.44)	–107.3 (–111.6, –103.0)
+ individual covariates^*b*^, neighborhood percent visible minority (fully adjusted)	0.96 (0.93, 0.99)	1.01 (0.94, 1.08)	1.04 (1.01, 1.07)	–20.5 (–24.7, –16.4)
24 cities (fully adjusted)	0.80 (0.75, 0.86)	0.98 (0.87, 1.10)	0.99 (0.93, 1.04)	–20.2 (–27.7, –12.6)
Quebec (fully adjusted)	0.90 (0.84, 0.96)	0.98 (0.86, 1.12)	1.03 (0.97, 1.09)	–16.1 (–26.5, –5.7)
^***a***^Census dissemination area proportion of individuals ≥ 15 years of age who were unemployed (preterm birth model only), proportion of individuals ≥ 15 years of age in the lowest income quintile, and proportion of females ≥ 25 years of age with postsecondary education. ^***b***^Maternal age and marital status, parity, urban/rural place of residence, airshed of maternal place of residence, place of birth of mother (within/outside Canada), year of birth, season of birth, and proportion of census dissemination area population who are visible minority; infant sex was also included in preterm birth, LBW, and birth weight models, and gestational age was also included in LBW and birth weight models.				

We also conducted sensitivity analyses employing data from ground-based monitors for 24 cities (see Supplemental Material, Table S4) based on 1,140,920 singleton live births, and employing data from Quebec only (*n* = 681,915 singleton live births), where we were able to adjust for individual-level maternal education as a covariate. Significant negative associations were observed with preterm birth in both instances (Table 2), which were larger in magnitude than those observed in the full data set, but associations with term LBW were null. Significant negative associations were observed with term birth weight, and a positive, nonsignificant association was observed with SGA in Quebec. These associations were comparable in magnitude with those observed in the full data set.

Table 4 summarizes associations stratified by maternal place of birth, urban/rural place of residence, neighborhood SES, and time period. For SGA there was a significant positive association among mothers born in Canada, but no association among mothers born elsewhere. Associations with term birth weight were negative for both groups, but the estimated association was stronger for mothers born in Canada. A nonsignificant positive association was observed with preterm birth in the 1999–2003 period, whereas there was a significant negative association during the 2004–2008 period. The association with LBW was negative and nonsignificant in 1999–2003 and positive and nonsignificant in 2004–2008. Significant negative associations were observed for term birth weight in both periods, but the magnitude of the association was larger in the 2004–2008 period.

**Table 4:** Associations between PM_2.5_ over the entire pregnancy and pregnancy outcome, by maternal place of birth, urban/rural place of residence, neighborhood SES, and time period, in models adjusted for neighborhood SES and individual covariates (per 10 μg/m^3^).

Interaction	Preterm birth OR (95% CI)	Term LBW OR (95% CI)	SGA OR (95% CI)	Term birth weight β (95% CI)
Maternal place of birth
Canada	0.94 (0.91, 0.98)	1.04 (0.96, 1.13)	1.09 (1.05, 1.13)	–30.9 (–35.6, –26.1)
Elsewhere	0.95 (0.88, 1.03)	0.97 (0.85, 1.11)	0.97 (0.92, 1.04)	–10.1 (–18.6, –1.6)
*p*-Value	0.81	0.38	0.0014	< 0.0001
Maternal place of residence
Urban	0.96 (0.92, 1.00)	1.00 (0.93, 1.08)	1.02 (0.99, 1.06)	–15.8 (–20.3, –11.3)
Rural	0.96 (0.88, 1.05)	0.97 (0.81, 1.17)	1.05 (0.97, 1.14)	–21.1 (–31.4, –10.9)
*p*-Value	1.00	0.76	0.52	0.35
Percent in lowest income quintile (age > 15 years)
1st tertile (≤ 9.25%)	0.92 (0.87, 0.98)	1.00 (0.88, 1.13)	1.02 (0.97, 1.08)	–17.8 (–24.2, –11.5)
2nd tertile (9.26–20.18%)	0.97 (0.91, 1.03)	0.92 (0.82, 1.04)	1.03 (0.97, 1.08)	–12.6 (–19.2, –6.1)
3rd tertile (> 20.18%)	0.97 (0.91, 1.03)	1.00 (0.89, 1.12)	0.98 (0.92, 1.03)	–9.4 (–17.8, –1.1)
*p*-Value	0.37	0.53	0.42	0.26
Period
1999–2003	1.05 (0.99, 1.11)	0.89 (0.80, 1.00)	1.03 (0.98, 1.08)	–12.5 (–18.6, –6.4)
2004–2008	0.90 (0.85, 0.94)	1.09 (0.99, 1.19)	1.05 (1.01, 1.10)	–28.7 (–34.0, –23.4)
*p*-Value	< 0.0001	0.006	0.56	< 0.0001

## Discussion

We employed estimates of PM_2.5_ exposure from a national spatiotemporal model in order to examine associations with preterm birth and term birth weight, LBW, and SGA in Canada between 1999 and 2008. Associations between PM_2.5_ and pregnancy outcomes were sensitive to adjustment for individual covariates, but not for neighborhood SES. In fully adjusted models, a 10-μg/m^3^ increase in PM_2.5_ over the entire pregnancy was associated with SGA [odds ratio (OR) = 1.04; 95% CI: 1.01, 1.07] and reduced term birth weight (OR = –20.5 g; 95% CI: –24.7, –16.4). Expressed per IQR (3.58 μg/m^3^) increase, these values are 1.014 (95% CI: 1.003, 1.026) and –7.4 g (95% CI: –8.9, –5.9).

To our knowledge, only three previous studies have employed model/satellite-based estimates of PM_2.5_ exposure to examine pregnancy outcomes in large studies examining both urban and rural areas. Kloog et al. (2012) reported that a 10-μg/m^3^ increase in PM_2.5_ was associated with a decrease in term birth weight of –13.8 g (95% CI: –21.10, –6.05) and an odds ratio for preterm birth of 1.06 (95% CI: 1.01, 1.13) from a study in Massachusetts, adjusting for infant and maternal characteristics including smoking. They found a positive, significant association with preterm birth based on entire pregnancy exposure, and a null association based on exposure during the last month. Hyder et al. (2014) estimated somewhat stronger associations except for preterm birth in a study in Connecticut and Massachusetts, also based on entire pregnancy exposure. They estimated that a 10-μg/m^3^ increase in PM_2.5_ was associated with decreases in birth weight ranging from –24.9 g (95% CI: –33.2, –20.8) to –78.8 g (95% CI: –95.4, –62.2) depending on the method of exposure assignment; odds ratios for term LBW ranged from 1.04 (95% CI: 0.92, 1.18) to 1.38 (95% CI: 1.04, 1.85), for SGA ranging from 1.13 (95% CI: 1.04, 1.18) to 1.38 (95% CI: 1.18, 1.54); and for preterm birth they ranged from 1.00 (95% CI: 0.96, 1.09) to 0.96 (95% CI: 0.81, 1.13). Associations were adjusted for infant and maternal characteristics (including smoking) and were consistently larger based on satellite-derived exposures than those based on ground-based monitoring, except for associations with preterm birth, which were consistently null. Greater exposure misclassification using ground-based monitoring versus satellite observations was identified as a possible explanation. Fleischer et al. (2014) reported a study of the association of satellite-based estimates of PM_2.5_ and preterm birth and LBW (all gestational ages) using the World Health Organization Global Survey on Maternal and Perinatal Health in Africa, Asia, and Latin America. PM_2.5_ was not associated with either preterm birth or LBW across the entire sample, but the highest quartiles of exposure were associated with LBW, and in China, the highest quartiles were associated with both preterm birth and LBW. Maternal smoking data were not available in this study.

Our results are consistent with those observed in a recent meta-analysis (Stieb et al. 2012), as well as a multi-center coordinated analysis (Dadvand et al. 2013). On the basis of a meta-analysis of case–control and cohort studies, Stieb et al. (2012) reported that a 10-μg/m^3^ increase in PM_2.5_ averaged over the entire pregnancy was associated with a summary odds ratio of 1.05 (95% CI: 0.99, 1.12) for LBW (*n* = 6 studies including studies of term LBW and all gestational-age LBW) as well as a summary –23.4 g (95% CI: –45.5, –1.4) reduction in birth weight (*n* = 7 studies including studies of term LBW and all gestational-age LBW). An increase of 10 μg/m^3^ PM_2.5_ averaged over the entire pregnancy was also associated with a summary odds ratio of 1.16 (95% CI: 1.07, 1.26) for preterm birth (*n* = 4 studies). Dadvand et al. (2013) reported that an increase of 10 μg/m^3^ PM_2.5_ was associated with an odds ratio of 1.04 (95% CI: 0.99, 1.09) for LBW and an –8.9 g reduction in birth weight (95% CI: –13.2, –4.6). They also found that associations were sensitive to adjustment for SES and maternal and infant characteristics.

Subgroup analyses in our study revealed that associations varied across subgroups based on maternal place of birth and on time period. For SGA there was a significant positive association among mothers born in Canada, but no association among mothers born elsewhere. Associations with term birth weight were negative for both groups, but the estimated association was stronger for mothers born in Canada. Factors accounting for greater vulnerability to effects of PM_2.5_ on risk of adverse pregnancy outcomes in population subgroups remain to be identified. Most other studies have examined effect modification by race rather than maternal place of birth, and results have been inconsistent (Bell et al. 2007; Morello-Frosch et al. 2010). A “healthy immigrant effect” has been suggested, in which recent immigrants experience better health and have better health behaviors (Ali et al. 2004). In our study, however, both PM_2.5_ exposure and prevalence of LBW and SGA were higher among non-Canadian–born mothers. Our observation of a weaker association for births to mothers not born in Canada would suggest greater “resistance” to the incremental effect of air pollution on these outcomes in this population, but this would need to be replicated in other studies. It has been suggested that lower SES confers a “double jeopardy” of increased stressors and increased exposure to environmental contaminants (Morello-Frosch and Shenassa 2006). Woodruff et al. (2003) reported disparities in air pollution exposure during pregnancy based on a multi-pollutant index by race but not educational attainment in the United States, and Buzzelli and Jerrett (2007), in a study in Toronto, found higher nitrogen dioxide exposures among both those with lower incomes but also higher-status occupations. We found similarly opposing trends in that PM_2.5_ exposure was higher in the highest tertile of percent low income, but there was also a trend of higher PM_2.5_ exposure with increasing percent of females who completed postsecondary education. Findings regarding effect modification by SES in other studies have been mixed (Morello-Frosch et al. 2010; Ponce et al 2005; Yi et al. 2010). In particular, in a study in Montreal, Canada, Généreux et al. (2008) found that associations between proximity to highways and pregnancy outcome were observed only among high-SES mothers. We observed a nonsignificant positive association with preterm birth in the 1999–2003 period, and a significant negative association in the 2004–2009 period, as well as significant negative associations with birth weight in both periods. PM_2.5_ concentrations declined between 1999 and 2008, but the prevalence of pregnancy outcomes did not exhibit a clear trend. Factors that could account for a change in risk associated with PM_2.5_ over time, particularly in the opposite direction for preterm birth and birth weight outcomes, require further exploration.

We found that estimated maternal exposures for the entire pregnancy were highly correlated with those for trimester and month of gestation, making it difficult to uniquely identify critical exposure windows. Nonetheless, for SGA and term birth weight, we found that late pregnancy exposures exhibited the largest associations. Consistent negative associations were observed between preterm birth and PM_2.5_ in most exposure periods. We do not consider it biologically plausible that air pollution exposure would have a protective effect with respect to preterm birth, and hypothesize that this may reflect bias or residual confounding. Results in previous studies have been mixed, although in a recent meta-analysis a significant summary odds ratio > 1 was reported for PM_2.5_ and preterm birth (*n* = 4 primary studies), and a nonsignificant summary odds ratio > 1 was reported for PM_10_ and preterm birth (*n* = 3 primary studies), both based on entire pregnancy exposure (Stieb et al. 2012). Results for individual trimesters were variable, including summary odds ratios > 1 and < 1, significant and nonsignificant (Stieb et al. 2012). In contrast, summary odds ratios for LBW were consistently > 1, and summary estimates of changes in birth weight were consistently negative across individual trimesters and entire pregnancy exposure, although their magnitude was larger based on entire pregnancy exposure (Stieb et al. 2012). The latter finding could be partly attributable to a scaling effect in that there tends to be less variability in exposures over the entire pregnancy than in shorter gestational periods, as we observed. Additional analysis of preterm birth examining effects of exposure in the days or weeks preceding birth using time-series or case-crossover methods may be informative.

Strengths of our study include the very large sample size, availability of exposure estimates for births across the entire country, and evaluation of associations at comparatively low levels of exposure. To our knowledge, this is one of the largest reported analyses of air pollution and pregnancy outcomes, nearly as large as the entire pooled ICAPPO multi-center study (Dadvand et al. 2013). The availability of exposure estimates from a nationally comprehensive model allowed us to include rural areas that would be excluded from studies relying on ground-based monitoring networks. Mean levels of PM_2.5_ exposure in our study were substantially lower than all centers included in the ICAPPO analysis other than Vancouver, allowing us to evaluate whether associations could be detected at low levels of exposure.

The study also has several limitations. We lacked data on potentially important confounding factors such as maternal smoking and alcohol use. However, it has been reported that associations between air pollution and preterm birth were not sensitive to adjustment for these factors in a case–control study of approximately 2,500 births (nested within a cohort of approximately 60,000) in 2003 in Los Angeles County (Ritz et al. 2007). In a national U.S. study of infant mortality based on approximately 2.5 million births between 1999 and 2002, associations with air pollution (including PM_2.5_) were not sensitive to adjustment for maternal smoking (Darrow et al. 2006). Villeneuve et al. (2011) reported that remote sensing–based estimates of PM_2.5_ exposure were negatively associated with smoking prevalence both in Ontario alone and in the rest of Canada, resulting in negative confounding of the association between PM_2.5_ and lung cancer and heart disease mortality (i.e., increased magnitude of association with PM_2.5_ after adjustment for smoking). They proposed a method for upward adjustment of air pollution relative risks derived from studies lacking individual data on smoking. Smoking during pregnancy was also strongly associated with maternal education in Quebec (Gilbert et al. 2014) and neighborhood SES in Alberta (Wood et al. 2014); thus, including SES variables as covariates may partially account for effects of smoking. We employed neighborhood-level data on SES. Luo et al. (2006), using data from Quebec, reported that individual measures of SES (maternal education) and neighborhood SES (low income) were independently associated with risk of preterm birth and SGA, although associations of individual-level maternal education were larger. Results of our sensitivity analysis using data from Quebec only, where we were able to adjust for individual-level maternal education, were consistent with national findings, suggesting that adjusting for neighborhood SES adequately controlled for confounding by SES.

## Conclusions

This study based on approximately 3 million births across Canada and employing PM_2.5_ estimates from a national spatiotemporal model provides further evidence linking PM_2.5_ and pregnancy outcomes. Associations between PM_2.5_ and pregnancy outcomes were sensitive to adjustment for individual covariates, but not neighborhood SES. In fully adjusted models, PM_2.5_ was associated with SGA and term birth weight. These associations varied across subgroups based on maternal place of birth and time period. Further study to identify population groups at greater risk and to examine mechanisms that could account for increased vulnerability would be desirable.

## Supplemental Material

(4.1 MB) PDF
