# Supplementary material for: Associations of Pregnancy Outcomes and PM2.5 in a National Canadian Study
Source: Environ Health Perspect. 2015 Jun 19;124(2):243–9. doi: 10.1289/ehp.1408995 (PMC4749076; doi:10.1289/ehp.1408995)
Supplement: (4.1 MB) PDF [file ehp.1408995.s001.acco.pdf]

Environ Health Perspect
DOI: 10.1289/ehp.1408995

Note to Readers: *EHP* strives to ensure that all journal content is accessible to all readers. However, some figures and Supplemental Material published in *EHP* articles may not conform to 508 standards due to the complexity of the information being presented. If you need assistance accessing journal content, please contact ehp508@niehs.nih.gov. Our staff will work with you to assess and meet your accessibility needs within 3 working days.

Supplemental Material

Associations of Pregnancy Outcomes and $PM_{2.5}$ in a National Canadian Study

David M. Stieb, Li Chen, Bernardo S. Beckerman, Michael Jerrett, Daniel L. Crouse, D. Walter Rasugu Omariba, Paul A. Peters, Aaron van Donkelaar, Randall V. Martin, Richard T. Burnett, Nicolas L. Gilbert, Michael Tjepkema, Shiliang Liu, and Rose M. Dugandzic

Table of Contents

Table S1. Summary model estimates for $PM_{2.5}$ land use regression comparing Canadian and previously published US models.

Figure S1. Cross-validation plot of predicted on observed $PM_{2.5}$ concentrations based on 1436 (10%) randomly selected leave-out observations from 22 monitoring sites. These data were used in cross validation only, not in modeling. Removal of eight cross validation data points over 35 μg/m^3 increased CV R2 from 0.36 to 0.53.

Figure S2. Provinces (top panel) and airsheds (bottom panel).

Table S2. Spearman correlations[a] among estimated $PM_{2.5}$ exposures by gestational period.

Table S3a. Individual years of maternal education vs. dissemination area percent post-secondary education (Quebec, females 25+).

Table S3b. Individual years of maternal education vs. dissemination area percent lowest income quintile (Quebec, age 15+).

Table S3c. Individual years of maternal education vs. dissemination area percent unemployed (Quebec, age 15+).

Table S4. Descriptive summary of monthly average $PM_{2.5}$ from ground-based monitoring data (without spatiotemporal modelling), 24 cities, 1999-2008 ($\mu g/m^{3.}$)

References

Table S1. Summary model estimates for $PM_{2.5}$ land use regression comparing Canadian and previously published US models.

**Variable**	**Coefficient**	**Std Error**	**z**	**P-value**
Canadian model:				
Remote Sensing $PM_{2.5}$: squared	0.072	2.70E-03	26.58	<0.0001
Remote Sensing $PM_{2.5}$: cubed	-2.51E-03	1.20E-04	-20.83	<0.0001
Remote Sensing *Canada Indicator	-0.18	0.018	-10.25	<0.0001
Open space within 200 m [acres]: squared	-1.91E-03	2.45E-04	-7.80	<0.0001
Intercept	6.874	0.169	40.60	<0.0001
US Model (Beckerman et al. 2013)				
Remote Sensing $PM_{2.5}$: squared	0.0701	3.0E-03	23.37	<0.0001
Remote Sensing $PM_{2.5}$: cubed	-2.4E-03	1.31E-04	-18.69	<0.0001
Developed land within 200 m [acres]	0.0404	0.0061	6.62	<0.0001
Intercept	5.9251	0.2222	26.66	<0.0001

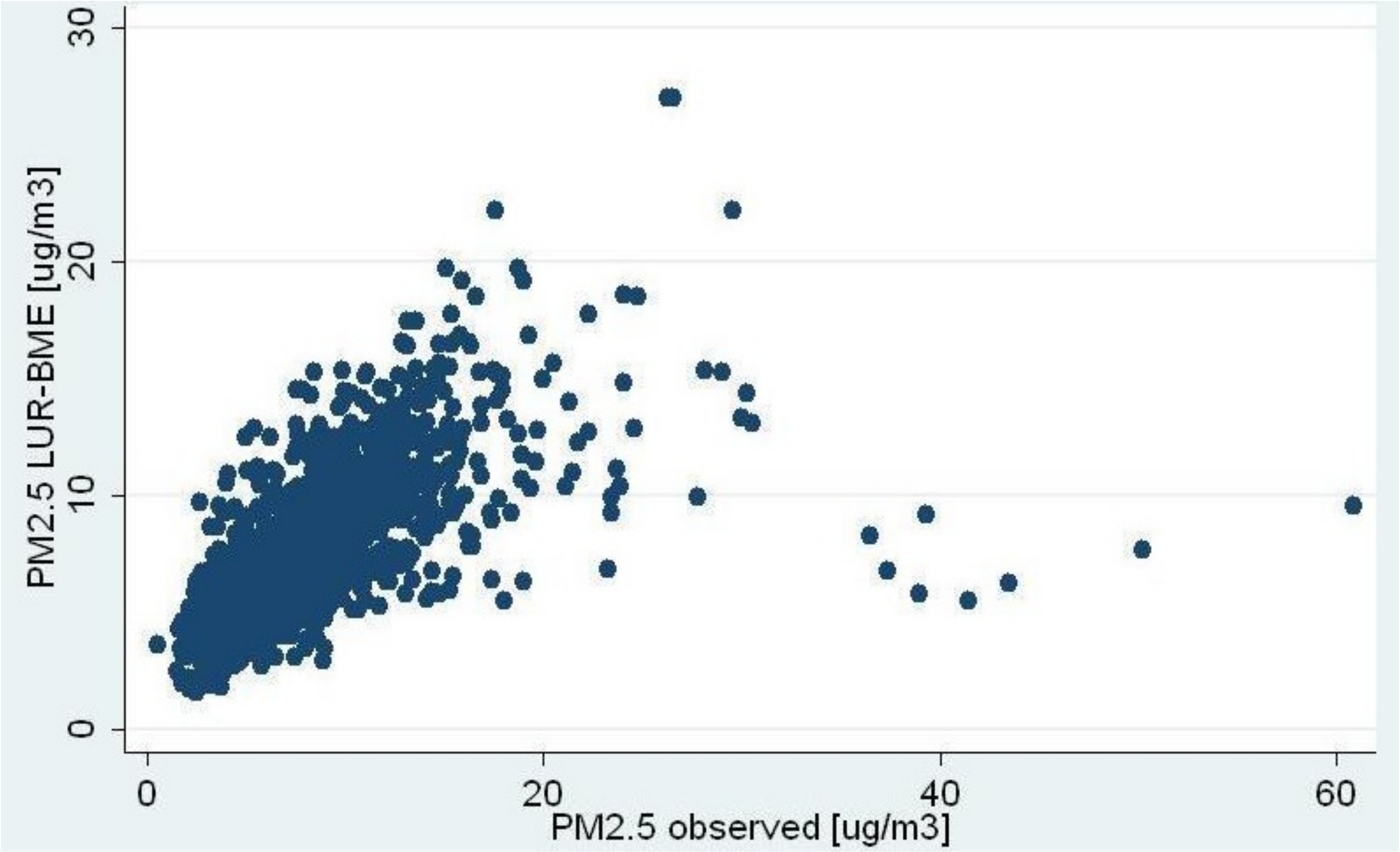

Figure S1. Cross-validation plot of predicted on observed $PM_{2.5}$ concentrations based on 1436 (10%) randomly selected leave-out observations from 22 monitoring sites. These data were used in cross validation only, not in modeling. Removal of eight cross validation data points over 35 $\mu g/m^3$ increased CV R2 from 0.36 to 0.53.

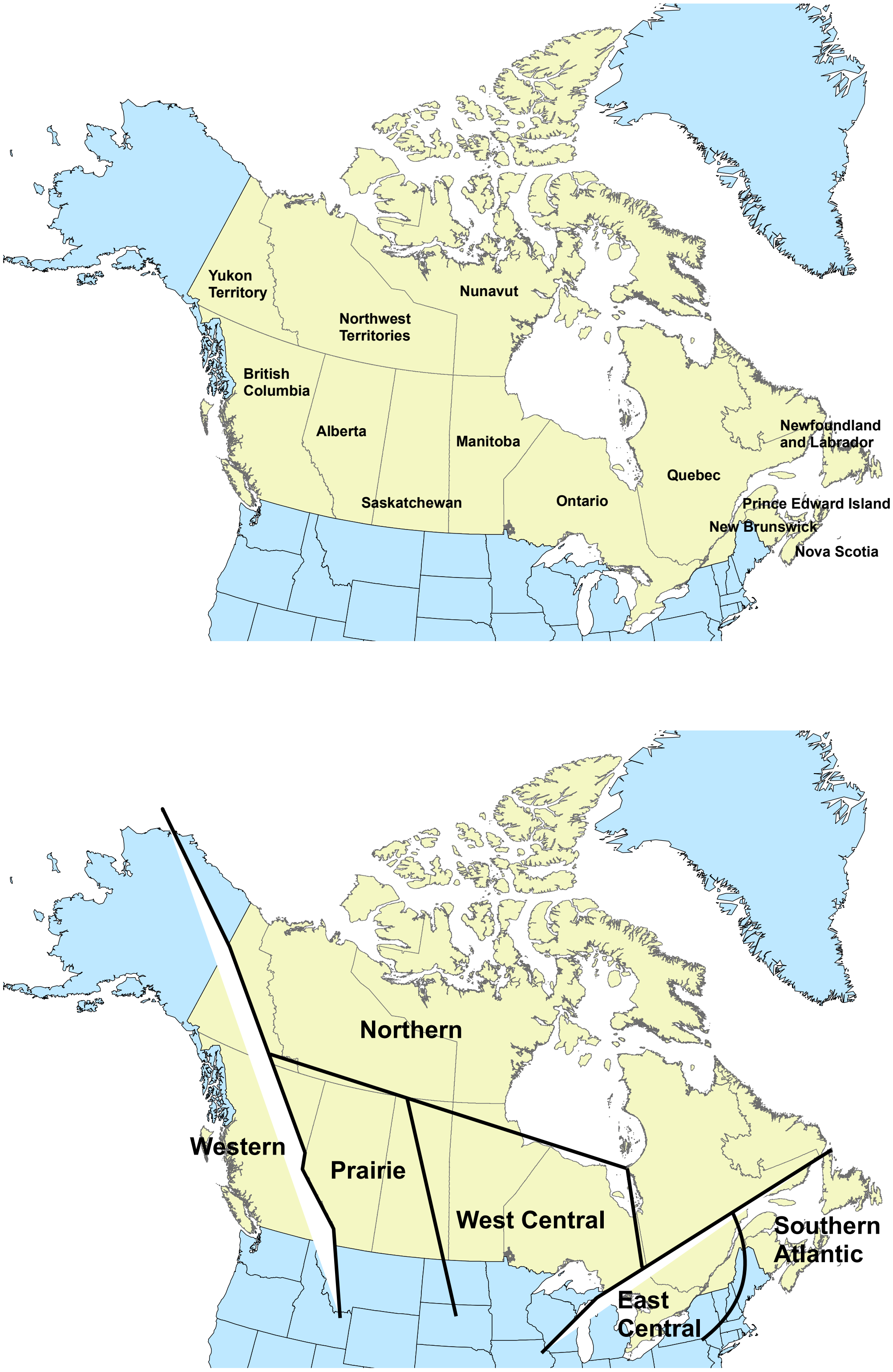

Figure S2. Provinces (top panel) and airsheds (bottom panel).

Table S2. Spearman correlations[a] among estimated $PM_{2.5}$ exposures by gestational period.

Period	Entire pregnancy	Last 30 days	Trimester 1	Trimester 2	Trimester 3	Month 1	Month 2	Month 3	Month 4	Month 5	Month 6	Month 7	Month 8	Month 9
Entire pregnancy	1.00	0.74	0.86	0.91	0.85	0.74	0.78	0.81	0.82	0.83	0.82	0.80	0.78	0.75
Trimester 1			1.00	0.70	0.57	0.88	0.95	0.88	0.72	0.62	0.57	0.53	0.50	0.51
Trimester 2				1.00	0.70	0.56	0.62	0.71	0.88	0.95	0.89	0.72	0.62	0.58
Trimester 3					1.00	0.51	0.50	0.53	0.57	0.62	0.71	0.87	0.95	0.90
Month 1						1.00	0.80	0.61	0.54	0.52	0.47	0.43	0.46	0.50
Month 2							1.00	0.80	0.61	0.55	0.53	0.47	0.43	0.46
Month 3								1.00	0.80	0.61	0.55	0.53	0.48	0.43
Month 4									1.00	0.80	0.62	0.55	0.53	0.48
Month 5										1.00	0.80	0.61	0.55	0.54
Month 6											1.00	0.80	0.61	0.55
Month 7												1.00	0.80	0.61
Month 8													1.00	0.80
Month 9														1.00

[a]p<0.0001 for all correlations.

Table S3a. Individual years of maternal education vs. dissemination area percent post-secondary education (Quebec, females 25+).

		Dissemination area percent of females who completed postsecondary education (age 25+)			
		1st tertile (≤20.36%)	2nd tertile (20.37-28.47%)	3rd tertile (>28.47%)	Total
Years of maternal education n[a] (row %)	≤11	102,515 (65.0)	38,660 (24.5)	16,590 (10.5)	157,765
	12-13	51,010 (55.0)	26,565 (28.6)	15,185 (16.4)	92,760
	≥14	145,855 (39.6)	112,355 (30.5)	109,820 (29.8)	368,030

Table S3b. Individual years of maternal education vs. dissemination area percent lowest income quintile (Quebec, age 15+).

		Dissemination area percent in lowest income quintile (age 15+)			
		1st tertile (≤9.25%)	2nd tertile (9.26-20.18%)	3rd tertile (>20.18%)	Total
Years of maternal education n[a] (row %)	≤11	33,155 (21.0)	51,390 (32.6)	73,215 (46.4)	157,760
	12-13	22,930 (24.7)	33,350 (35.9)	36,485 (39.3)	92,765
	≥14	124,170 (33.7)	133,470 (36.3)	110,390 (30.0)	368,030

Table S3c. Individual years of maternal education vs. dissemination area percent unemployed (Quebec, age 15+).

		Dissemination area percent unemployed (age 15+)			
		1st tertile (≤4.6%)	2nd tertile (4.61-8.22%)	3rd tertile (>8.22%)	Total
Years of maternal education n[a] (%)	≤11	38,385 (24.3)	48,785 (30.9)	70,595 (44.7)	157,765
	12-13	24,775 (26.7)	31,360 (33.8)	36,625 (39.5)	92,760
	≥14	124,480 (33.8)	126,175 (34.3)	117,370 (31.9)	368,025

[a]In accordance with Statistics Canada disclosure rules, all frequencies were randomly rounded to base five; percentages are based on unrounded data.

Table S4. Descriptive summary of monthly average $PM_{2.5}$ from ground-based monitoring data (without spatiotemporal modelling), 24 cities, 1999-2008 ($\mu g/m^{3}$)

City (census subdivision)	2006 Population	Mean	SD[a]	5th %ile	95th %ile	IQR[b]
St. John's	100,646	6.1	2.2	3.1	10.7	2.9
Saint John	68,043	6.9	3.0	3.9	11.6	3.1
Fredericton	50,535	5.9	2.1	2.9	9.3	3.2
Quebec	491,142	10.2	3.9	5.6	15.8	4.4
Trois-Rivieres	126,293	9.7	2.8	5.9	14.1	4.0
Montreal	1,620,693	11.2	4.0	6.4	18.9	4.7
Ottawa	812,129	8.6	2.6	4.7	12.2	3.5
Oshawa	141,590	9.8	2.6	5.8	13.9	3.7
Toronto	2,503,281	10.9	2.6	7.2	15.3	3.7
St. Catharines	131,989	10.2	2.7	5.8	14.7	3.7
Hamilton	504,559	11.6	2.7	7.5	16.1	3.8
Mississauga	668,599	10.1	2.4	6.1	13.9	3.6
Brampton	433,806	9.8	2.5	5.7	13.6	3.1
Kitchener	204,668	10.0	3.0	5.7	15.3	3.3
Windsor	216,473	11.5	2.8	7.5	16.2	3.8
Winnipeg	633,451	7.2	2.1	4.4	10.3	2.8
Calgary	988,812	9.0	3.3	4.7	13.9	5.1
Edmonton	730,372	9.1	3.7	4.4	15.3	5.5
Kelowna	107,035	6.6	3.2	3.0	10.6	3.5
Kamloops	80,376	6.6	3.1	3.4	11.1	3.8
Richmond	174,461	6.5	2.1	4.2	10.3	2.6
Vancouver	578,041	7.0	1.9	4.4	10.5	2.4
Nanaimo	78,692	5.2	1.8	2.9	8.1	2.7
Victoria	78,057	7.1	2.5	3.8	11.7	3.3
Total/ Population weighted average	11,523,743	9.7				3.9

[a]standard deviation; [b]interquartile range

References

Beckerman BS, Jerrett M, Serre M, Martin RV, Lee SJ, van Donkelaar A, et al. 2013. A hybrid approach to estimating national scale spatiotemporal variability of $PM_{2.5}$ in the contiguous United States. Environ Sci Technol 47:7233-7241.
